# Variational quantum metrology for multiparameter estimation under dephasing noise

**DOI:** 10.1038/s41598-023-44786-0

**Published:** 2023-10-18

**Authors:** Trung Kien Le, Hung Q. Nguyen, Le Bin Ho

**Affiliations:** 1 Department of Physics, University of California, Santa Barbara, Santa Barbara, USA; 2 Present Address: Department of Applied Physics, Stanford University, Stanford, California 94305 USA; 3 Nano and Energy Center, University of Science, Vietnam National University, Hanoi, 120401 Vietnam; 4 Frontier Research Institute for Interdisciplinary Sciences, Tohoku University, Sendai, 980-8578 Japan; 5 Department of Applied Physics, Graduate School of Engineering, Tohoku University, Sendai, 980-8579 Japan

**Keywords:** Quantum information, Quantum mechanics, Quantum metrology, Quantum simulation, Qubits

## Abstract

We present a hybrid quantum-classical variational scheme to enhance precision in quantum metrology. In the scheme, both the initial state and the measurement basis in the quantum part are parameterized and optimized via the classical part. It enables the maximization of information gained about the measured quantity. We discuss specific applications to 3D magnetic field sensing under several dephasing noise models. Indeed, we demonstrate its ability to simultaneously estimate all parameters and surpass the standard quantum limit, making it a powerful tool for metrological applications.

## Introduction

Quantum metrology is an estimation process that utilizes unique quantum phenomena such as entanglement and squeezing to improve the precision of estimation beyond classical limits^1–3^. Recent development in quantum computing leads to numerous optimal algorithms for enhancing precision in single-parameter estimation, such as adaptive measurements^4–7^, quantum error correction^8,9^, and optimal quantum control^10–12^. So far, a variational algorithm has been demonstrated by combining the advantages of both quantum and classical systems for quantum-enhanced metrology^12–15^. A similar protocol for spin systems was also introduced^16,17^.

Multiparameter estimation is essential in various fields, such as Hamiltonian tomography^18^, multiphase sensing^19–21^, gravitational wave detection^22^, and atomic clocks^23,24^. However, the estimation is more challenging due to the incompatibility^25^. In these cases, simultaneous determination of all parameters is impossible, resulting in a tradeoff^26^. Numerous techniques have been introduced to tackle this challenge, including establishing optimal measurement strategies^27,28^, employing parallel scheme^21^, sequential feedback scheme^29^, and implementing post-selection procedures^30^. More recently, a variational toolbox for multiparameter estimation was proposed^31^, which is a generalization from the previous work mentioned above^14^.

While using variational schemes is promising, their potential significance in multiparameter quantum metrology has yet be fully understood, even in principle. Furthermore, determining the optimal quantum resources and measurement strategy to extract maximum information about all parameters is limited by the tradeoffs in estimating incompatible observables^26,32,33^ and required collective measurements over multiple copies of a probe state^32,33^. Therefore, finding a suitable and practical strategy for precise estimation of multiple parameters remains a thriving area of quantum metrology.

In this work, we propose a variational scheme to enhance the precision of multiparameter estimation in the presence of dephasing noise. The basic idea is to use a quantum computer to prepare a trial state (an ansatz) that depends on a set of trainable variables. The state is subjected to a series of control operations, representing unknown multiparameter and noise, and then is measured through observables determined by other trainable variables. The measurement results are used to update the trainable variables and optimize the estimation of the unknown parameters.

Optimizing both the initial probe state and the measurement operators allows us to identify suitable conditions for the quantum probe to increase sensitivity and achieve the ultimate quantum limit for all parameters. In numerical simulations, we estimate a 3D magnetic field under a dephasing noise model and find that sensitivity for all parameters can simultaneously reach the ultimate quantum bound, i.e., the classical bound equals the quantum bound. We also examine a time-dependent Ornstein-Uhlenbeck model^34^ and observe results surpassing the standard quantum limit by increasing the probe’s number of particles. This approach holds promise for a wide range of metrological applications, including external field sensing, precision spectroscopy, gravitational wave detection, and others, where the effects of noises cannot be ignored.

**Figure 1:**
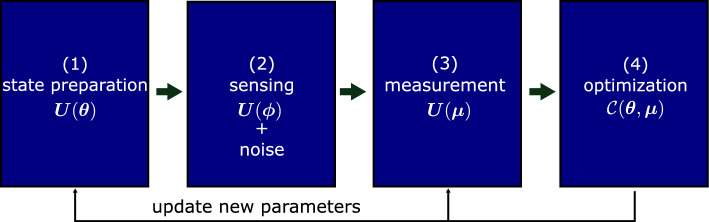
Variational quantum metrology. (1) use quantum circuit $\varvec{U}(\varvec{\theta })$ to prepare a variational state; (2) encode multiparameter $\varvec{\phi }$ and noise using $\varvec{U}(\varvec{\phi })$ and noise channels; (3) use circuit $\varvec{U}(\varvec{\mu })$ to create a variational POVM for measurement; (4) send measurement results to a classical computer to optimize cost function $\mathcal {C}(\varvec{\theta },\varvec{\mu })$ using a gradient-based optimizer. Update new training variables and repeat the scheme until it converges.

## Results

### Variational quantum metrology

The goal of multiparameter estimation is to evaluate a set of unknown *d* parameters $\varvec{\phi }= (\phi _1, \phi _2, \ldots , \phi _d)^\intercal$, which are imprinted onto a quantum probe via a unitary evolution $\varvec{U}(\varvec{\phi }) = \exp (-it\varvec{H}\varvec{\phi }) = \exp (-it\sum _{k=1}^dH_k\phi _k)$, where $\varvec{H} = (H_1, H_2, \ldots , H_d)$ are non-commuting Hermitian Hamiltonians. The precision of estimated parameters $\check{\phi }$ is evaluated using a mean square error matrix (MSEM) $V = \sum _m p(m|\varvec{\phi }) \big [\varvec{\check{\phi }}(m)-\varvec{\phi }\big ] \big [\varvec{\check{\phi }}(m)-\varvec{\phi }\big ]^\intercal ,$ where $p(m|\varvec{\phi }) = \textrm{Tr}[\rho (\varvec{\phi }) E_m]$ is the probability for obtaining an outcome *m* when measuring the final state $\rho (\varvec{\phi })$ by an element $E_m$ in a positive, operator-value measure (POVM). For unbiased estimators, the MSEM obeys the Cramér-Rao bounds (CRBs)^35–38^

1 $$\begin{aligned} \textrm{Tr}\big [WV\big ] \ge \textsf {C}_\textsf {F} \ge \textsf {C}_{\textsf {NH}} \ge \textsf {C}_{\textsf {H}} \ge \textsf {C}_{\textsf {S}}, \end{aligned}$$

where *W* is a scalar weight matrix, which can be chosen as an identity matrix without loss of generality. The classical bound is $\textsf{C}_{\textsf{F}} = \textrm{Tr}[ {WF}^{-1}]$, where *F* is the classical Fisher information matrix (CFIM) with elements $F_{ij} = \sum _m \frac{1}{p(m|\varvec{\phi })} [\partial _{\phi _i} p(m|\varvec{\phi })] [\partial _{\phi _j} p(m|\varvec{\phi })]$^39^. The Nagaoka–Hayashi bound $\textsf{C}_{\textsf{NH}}$ and Holevo bound $\textsf{C}_{\textsf{H}}$ are given via semidefinite programming, i.e., $\textsf{C}_{\textsf{NH}} = \underset{\{D, X\}}{\min } \textrm{Tr}[(W\otimes \rho (\varvec{\phi }))\ \cdot D]$^38,40^, and $\textsf{C}_{\textsf{H}} = \underset{\{X\}}{\min } \big ( \textrm{Tr}[W\textrm{Re}Z+ \vert \vert \sqrt{W} \textrm{Im}Z\sqrt{W}]\vert \vert _1 \big )$^36,41^, where *D* is a *d*-by-*d* matrix contains Hermitian operators $D_{ij}$ and satisfies $D \ge XX^\intercal$, $X = (X_1, X_2,\ldots ,X_d)^\intercal$ satisfies $\textrm{Tr}[\rho (\varvec{\phi })X_j] = \phi _j$ and $\textrm{Tr}[X_i\partial _{\phi _j}\rho (\varvec{\phi })] =\delta _{ij}$, *Z* is a positive semidefinite matrix with elements $Z_{ij} = \textrm{Tr}[X_iX_j\rho (\varvec{\phi })]$. Finally, $\textsf{C}_{\textsf{S}} = \textrm{Tr}[WQ^{-1}]$ is a symmetric logarithmic derivative (SLD) quantum bound where $Q_{ij} = \textrm{Re} \big [\textrm{Tr}[\rho (\varvec{\phi })L_iL_j]\big ]$ is the real symmetric quantum Fisher information matrix (QFIM) that defined through the SLD $2\partial _{\phi _j}\rho (\varvec{\phi }) = \{L_j,\rho (\varvec{\phi })\}$^39^.

Although optimal estimators can achieve $\textsf{C}_{\textsf{F}}$^42^, the $\textsf{C}_{\textsf{NH}}$ can be attainted with separable measurements for qubits probes^40^, and asymptotic achievement of $\textsf{C}_{\textsf{H}}$ is possible^43–47^, it is not always possible to attain $\textsf{C}_{\textsf{S}}$ for multiparameter estimation^41^. In this work, we attempt to reach this bound. In some instances, $\textsf{C}_{\textsf{H}} = \textsf{C}_{\textsf{S}}$ if a weak commutativity condition $\mathrm{Im(Tr}[L_jL_i\rho (\varvec{\phi })]) = 0$ is met^41,48^. A similar condition for pure states is also applied to attain $\textsf{C}_{\textsf{F}} =\textsf{C}_{\textsf{S}}$^19,49,50^. Further discussion on the interplay between $\textsf{C}_{\textsf{NH}}, \textsf{C}_{\textsf{H}},$ and $\textsf{C}_{\textsf{S}}$ has been reported^37^. However, this condition alone is insufficient to achieve the quantum bounds practically; instead, attaining $\textsf{C}_{\textsf{S}}$ and $\textsf{C}_{\textsf{H}}$ also requires entangled measurements (POVM) over multiple copies^45,46^. Recently, Yang et al., have derived saturation conditions for general POVMs^28^. To be more precise, when $F \ge Q > 0$ and for any arbitrary full-rank positive weight matrix $W > 0$, the equally $\textsf{C}_{\textsf{F}} = \textsf{C}_{\textsf{S}}$ implies $F = Q$.

This paper presents a variational quantum metrology (VQM) scheme following Meyer et al. toolbox^31^ as sketched in Fig. 1 to optimize both the preparation state and POVM. A quantum circuit $\varvec{U}(\varvec{\theta })$ is used to generate a variational preparation state with trainable variables $\varvec{\theta }$. Similar quantum circuit with variables $\varvec{\mu }$ is used to generate a variational POVM $\varvec{E}(\varvec{\mu }) = \{E_m(\varvec{\mu }) = \varvec{U}^\dagger (\varvec{\mu })E_m \varvec{U}(\varvec{\mu }) > 0 \big | \sum _m E_m(\varvec{\mu }) = \varvec{I}\}$. Using classical computers, a cost function $\mathcal {C}(\varvec{\theta },\varvec{\mu })$ can be optimized to update the variables for quantum circuits, resulting in enhanced information extraction. The scheme is repeated until it converges.

**Figure 2:**
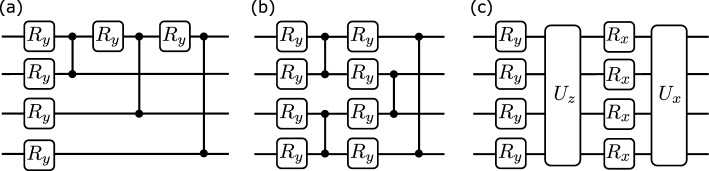
Ansatzes for preparation state and POVM. (**a**) Star topology entangled ansatz. (**b**) Ring topology entangled ansatz. (**c**) Squeezing ansatz. In the circuits, $R_{x(y)}$: *x*(*y*)-rotation gate, $U_{x(z)}$: global Mølmer–Sørensen gate, $\bullet \!{-}\!\bullet$: controlled-Z gate.

To investigate the attainable the ultimate SLD quantum bound, we define the cost function by a relative difference^47^

2 $$\begin{aligned} \mathcal {C} (\varvec{\theta }, \varvec{\mu }) = 1 - \dfrac{\textsf{C}_{\textsf{S}}}{\textsf{C}_{\textsf{F}}}, \end{aligned}$$

which is positive semidefinite according to Eq. (1). The variables are trained by solving the optimization task $\underset{\{\varvec{\theta }, \varvec{\mu }\}}{\arg \min }\ \mathcal {C} (\varvec{\theta }, \varvec{\mu })$. As the value of $\mathcal {C}(\varvec{\theta }, \varvec{\mu })$ approaches zero, we reach the ultimate SLD quantum bound where $\textsf{C}_{\textsf{F}}= \textsf{C}_{\textsf{S}}$. Notably, we strive for agreement between classical and SLD quantum bounds assuming $\textrm{Tr}[WV] = \textsf{C}_{\textsf{F}}$, and thus, omit discussion on the estimator for achieving $\textrm{Tr}[WV] = \textsf{C}_{\textsf{F}}$. Moreover, the cost function (2) serves as a technical tool to optimize the variational scheme, while the main analyzing quantities are CRBs. A vital feature of the VQM is using variational quantum circuits, which allows for optimizing the entangled probe state and measurements to extract the maximum information about the estimated parameters. This approach thus does not require entangled measurements over multiple copies. We further discuss various cost functions in the Discussion section.

### Ansatzes

We propose three variational circuits: a star topology ansatz, a ring topology ansatz, and a squeezing ansatz. The first two ansatzes are inspired by quantum graph states, which are useful resources for quantum metrology^51,52^. A conventional graph state is formed by a collection of vertices *V* and edges *D* as $G(V,D) = \prod _{{i,j} \in D} \textrm{CZ}^{ij} |+\rangle ^{V}$, where $\textrm{CZ}^{ij}$ represents the controlled-Z gate connecting the *i* and *j* qubits, and $|+\rangle$ is an element in the basis of Pauli $\sigma _x$. The proposed ansatzes here incorporate *y*-rotation gates ($R_y(\theta ) = e^{-i\theta \sigma _y/2}$) at every vertex prior to CZ gates (see Fig. 2a,b). The squeezing ansatz in Fig. 2c is inspired by squeezing states, which is another useful resource for quantum metrology^53–55^. It has *x*(*y*)-rotation gates and global Mølmer–Sørensen gates $U_{x(z)}$, where $U_{x(z)} = \exp (-i\sum _{j=1}^N\sum _{k = j+1}^N \sigma _{x(z)}\otimes \sigma _{x(z)} \frac{\chi _{jk}}{2})$ for an *N*-qubit circuit^56^. The trainable variables for one layer are $2N - 2$, 2*N*, and $N(N+1)$ for the star, ring, and squeezing ansatz, respectively. Hereafter, we use these ansatzes for generating variational preparation states and variational POVM in the VQM scheme.

**Figure 3:**
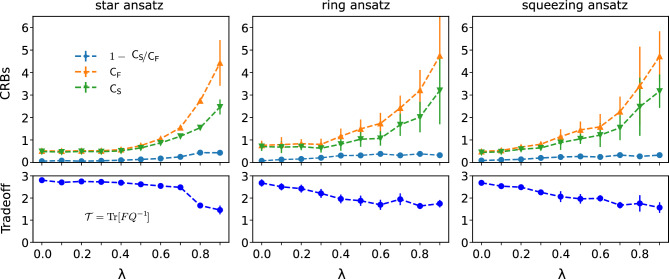
Variational quantum metrology under dephasing noise. (Top): plot of the optimal cost function $\mathcal {C} (\varvec{\theta }, \varvec{\mu })$, classical bound $\textsf{C}_{\textsf{F}}$, and SLD quantum bound $\textsf{C}_{\textsf{S}}$ as functions of dephasing probability. From left to right: star, ring, and squeezing ansatz. (Bottom): plot of corresponding tradeoff $\mathcal {T}$. Numerical results are calculated at $N = 3$, the optimal number of layers in Fig. 8, and the results are averaged after 10 samples.

### Multiparameter estimation under dephasing noise

After preparing a variational state $\rho (\varvec{\theta }) = \varvec{U}(\varvec{\theta }) \rho _0\varvec{U}(\varvec{\theta })$, we use it to estimate a 3D magnetic field under dephasing noise. The field is imprinted onto every single qubit via the Hamiltonian $\varvec{H} = \sum _{i \in \{x,y,z\}}\phi _i\sigma _i$, where $\varvec{\phi }= (\phi _x, \phi _y, \phi _z)$, and $\sigma _i$ is a Pauli matrix. Under dephasing noise, the variational state $\rho (\varvec{\theta })$ evolves to^13^

3 $$\begin{aligned} \mathcal {E}_t(\rho ) = \Bigg [\prod _{k=1}^N e^{\gamma t\mathcal {L}^{(k)}}\Bigg ] e^{-it\mathcal {H}}\rho , \end{aligned}$$

where we omitted $\varvec{\theta }$ in $\rho (\varvec{\theta })$ for short. The superoperator $\mathcal {H}$ generates a unitary dynamic $\mathcal {H}\rho = [\varvec{H}, \rho ]$, and $\mathcal {L}^{(k)}$ is a non-unitary dephasing superoperator with $\gamma$ is the decay rate. In terms of Kraus operators, the dephasing superoperator gives

4 $$\begin{aligned} e^{\gamma t\mathcal {L}^{(k)}}\rho = K_1^{(k)}\rho [K_1^{(k)}]^\dagger + K_2^{(k)}\rho [K_2^{(k)}]^\dagger , \end{aligned}$$

where $$K_1 = \begin{pmatrix} \sqrt{1-\lambda } &{} 0\\ 0&{}1\end{pmatrix}$$ and $$K_2 = \begin{pmatrix} \sqrt{\lambda } &{} 0\\ 0&{}0\end{pmatrix}$$ are Kraus operators, and $\lambda = 1-e^{-\gamma t}$ is the dephasing probability. Finally, the state is measured in the variational POVM $\varvec{E}(\varvec{\mu })$ and yields the probability $p(m) = \textrm{Tr}[\mathcal {E}_t(\rho )E_m(\varvec{\mu })]$. Note that *p* also depends on $\varvec{\theta }, \varvec{\phi }$, and $\varvec{\mu }$.

It is important to attain the ultimate SLD quantum bound, i.e., $\textsf{C}_{\textsf{F}} = \textsf{C}_{\textsf{S}}$. We thus compare numerical results for the cost function, $\textsf{C}_{\textsf{F}}$, and $\textsf{C}_{\textsf{S}}$ as shown in the top panels of Fig. 3. At each $\lambda$, the cost function and other quantities are plotted with the optimal $\theta$ obtained after stopping the training by EarlyStopping callback^57^. The numerical results are presented at $N = 3$, and the number of layers is chosen from their optimal values as shown in the Method and Fig. 8. Through the paper, we fixed $(\phi _x, \phi _y, \phi _z) = (\pi /6, \pi /6, \pi /6)$.

We find that for small noises, $\textsf{C}_{\textsf{F}}$ reaches $\textsf{C}_{\textsf{S}}$, which is consistent with earlier numerical findings^46,47^. Remarkably, different from the previous findings where the convergence of these bounds is not clear, here we show that both $\textsf{C}_{\textsf{F}}$ and $\textsf{C}_{\textsf{S}}$ remain small (also in comparison to previous work^31^) without any divergence. We further compare the performance of the star ansatz to that of the ring and squeezing ansatzes. It saturates the ultimate quantum limit for dephasing probabilities $\lambda < 0.5$, whereas the ring and squeezing ansatzes only reach the limit for $\lambda < 0.2$. The reason is that the star graph exhibits a central vertex connected to the remaining $N-1$ surrounding vertices, which facilitates robust quantum metrology, as discussed in^51^.

Furthermore, we evaluate the tradeoff between the CFIM and QFIM by introducing a function $\mathcal {T} = \text {Tr}[FQ^{-1}].$ For unknown *d* parameters, the naive bound is $\max (\mathcal {T}) = d$, leading to simultaneous optimization of all parameters. The results are shown in the bottom panels of Fig. 3 and agree well with the CRBs presented in the top panels, wherein $\mathcal {T}\rightarrow 3$ whenever the SLD quantum bound is reached. So far, we observe that $\mathcal {T}> d/2$ for all cases, which is better than the theoretical prediction previously^58^. This observation exhibits a practical advantage of the VQM approach across different levels of noise.

**Figure 4:**
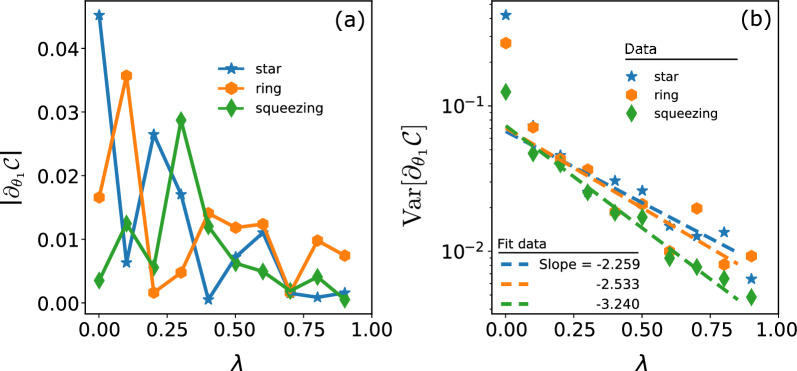
Barren plateau. (**a**) Plot of $|\partial _{\theta _1}\mathcal {C}|$ as a function of the dephasing probability $\lambda$. (**b**) Plot of the variance of gradient Var[$\partial _{\theta _1}\mathcal {C}$]. The slope of each fit line indicates the exponential decay of the gradient, which is a sign of the barren plateau effect. The results are taken average after 200 runs.

### Barren plateaus

Variational quantum circuits under the influence of noises will exhibit a barren plateau (BP), where the gradient along any direction of the variables’ space vanishes exponentially with respect to the noise level^59^. The BP prevents reaching the global optimization of the training space, thereby reducing the efficiency and trainability of the variational quantum circuit. However, BPs can be partially mitigated through carefully designing ansatzes and cost functions^60^.

The deviation of CRBs shown in Fig. 3 may be subject to the BP raised by noise. We examine such dependent and show the results in Fig. 4. We plot $|\partial _{\theta _1} \mathcal {C}|$ (Fig. 4a) and Var$[\partial _{\theta _1} \mathcal {C}]$ (Fig. 4b), where $\mathcal {C}$ is defined in Eq. (2) after 200 runs with random initialization of $\varvec{\theta }$ and $\varvec{\mu }$ for each value of $\lambda$. As predicted, both of them demonstrate an exponential decline with an increase in the dephasing probability. Especially, Var$[\partial _{\theta _1} \mathcal {C}]$ exponentially vanishes with the slope of -2.259, -2.533, and -3.240 for the star, ring, and squeezing ansatz, respectively. The star ansatz exhibits slower gradient decay as $\lambda$ approaches 1 due to its smaller trainable variables’ space than the ring and squeezing ansatz. This indicates better training and less susceptibility to vanishing gradients, leading to better achievement of the ultimate quantum bound.

**Figure 5:**
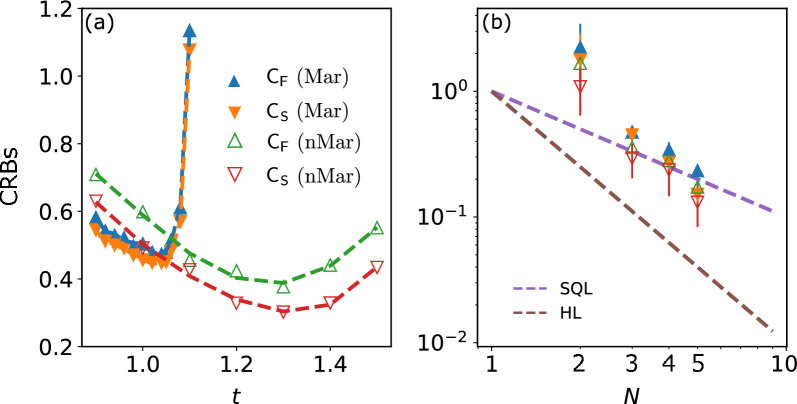
Variational quantum metrology under time-dephasing noise. (**a**) We present the CRBs as functions of the sensing time, demonstrating an optimal sensing time for achieving each minimum CRB. The non-Markovian dephasing (nMar) produces lower metrological bounds in comparison to the Markovian one (Mar). (**b**) Plot of the minimal bounds for cases in (**a**), comparing them with the standard quantum limit (SQL) and the Heisenberg limit (HL). For non-Markovian metrology, the bounds surpass the SQL, as predicted.

### Multiparameter estimation under the Ornstein–Uhlenbeck model

We consider the Ornstein-Uhlenbeck model, where the noise is induced by the stochastic fluctuation of the external (magnetic) field^34^. The Kraus operators are^61^

5 $$\begin{aligned} K_1(t) = \begin{pmatrix} \sqrt{1-q(t)} &{} 0 \\ 0 &{} 1 \end{pmatrix},\ K_2(t) = \begin{pmatrix} \sqrt{q(t)} &{} 0 \\ 0 &{} 0 \end{pmatrix} , \end{aligned}$$

where $q(t) = 1-e^{-f(t)}$ with $f(t) = \gamma [t+\tau _c (e^{-t/\tau _c}-1)]$, and $\tau _c$ represents the memory time of the environment. In the Markovian limit ($\tau _c\rightarrow 0$), $f(t) = \gamma t$, which corresponds to the previous dephasing case. In the non-Markovian limit with large $\tau _c$, such as $t/\tau _c\ll 1$, we have $f(t) = \frac{\gamma t^2}{2\tau _c}$. In the numerical simulation, we fixed $\gamma = 0.1$ and $\tau _c = 20$ (for non-Markovian)

6 $$\begin{aligned} q(t) = {\left\{ \begin{array}{ll} 1-\exp (- 0.1t) \;\;\;\; &{}\text {Markovian,} \\ 1-\exp (-\frac{t^2}{400}) \;\;\; &{}\text {non-Markovian.} \end{array}\right. } \end{aligned}$$

We use this model to study the relationship between sensing time, Markovianity, and ultimate attainability of the quantum bound. Figure 5a displays the optimal CRBs for Markovian and non-Markovian noises as functions of sensing time *t*. As previously reported in^20^, there exists an optimal sensing time that minimizes the CRBs for each case examined here. Moreover, the non-Markovian dephasing (nMar) results in lower metrological bounds as compared to the Markovian case (Mar). So far, the minimum CRBs for different *N* are presented in Fig. 5b. The results demonstrate that with an increase in *N*, the non-Markovian noise attains a better bound than the standard quantum limit (SQL) for both classical and SLD quantum bounds. This observation agrees with previous results reported using semidefinite programming^62^, indicating the potential of variational optimization for designing optimal non-Markovian metrology experiments. Finally, we note that in the Ornstein-Uhlenbeck model, the SLD quantum bound is unachievable, as indicated by $\textsf{C}_{\textsf{F}} > \textsf{C}_{\textsf{S}}$. It remains a question for future research on whether one can attain the SLD quantum bound $\textsf{C}_{\textsf{S}}$ with probe designs, and the existence of tight bounds in the non-Markovian scenario.

## Discussion

### Concentratable entanglement

We discuss how the three ansatzes create entangled states and the role of entangled resources in achieving the SLD quantum bound in VQM. We analyze entanglement using the concentratable entanglement (CE) defined by^63^

7 $$\begin{aligned} \xi (\psi ) = 1 - \dfrac{1}{2^{|s|}} \sum _{\alpha \in \mathcal {P}(s)} \textrm{Tr}[\rho ^2_\alpha ], \end{aligned}$$

where $\mathcal {P}(s)$ is the power set of $s, \forall s\in \{1,2,\ldots ,N\}$, and $\rho _\alpha$ is the reduced state of $|\psi \rangle$ in the subsystem $\alpha$ with $\rho _\emptyset := \varvec{I}$. Practically, $\xi (\psi )$ can be computed using the SWAP test circuit as stated in Ref.^63^, where $\xi (\psi ) = 1 - p(\varvec{0}),$ with $p(\varvec{0})$ is the probability of obtaining $|00\cdots 0\rangle$. The ability of the SWAP test to compute CE is due to the equivalence between conditional probability distribution and the definition of CE.

**Figure 6:**
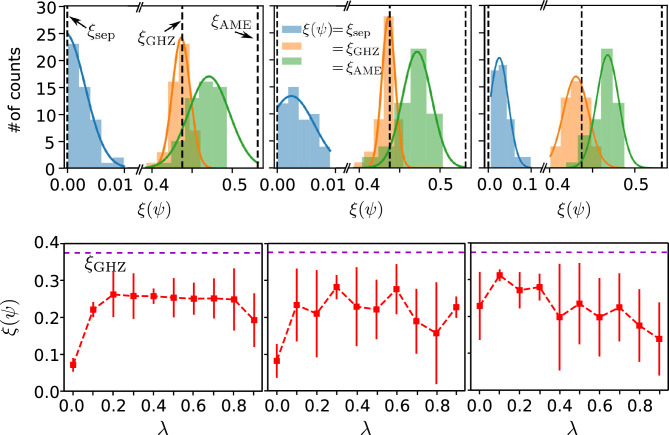
Entanglement generation. (Top): from left to right: the distribution of training CEs corresponds to the star, ring, and squeezing ansatzes, respectively. All the ansatzes can produce separable and GHZ states, but generating an AME state is challenging. The results are shown at $N=4$ and (2–2) layers for each ansatz. (Bottom): the CEs are ploted at the optimal CRBs in Fig. 3, using the same circuit setup that in the figure. Again, $\lambda$ is the dephasing probability.

We first train the three ansatzes to evaluate their ability of entangled-state generation. Particularly, the training process aims to generate quantum states with $\xi (\psi ) =\{ \xi _{\textrm{sep}}, \xi _{\textrm{GHZ}}, \xi _{\textrm{AME}} \}$, where $\xi _{\textrm{sep}} = 0$ for a separable state, $\xi _{\textrm{GHZ}} = \frac{1}{2} - \frac{1}{2^N}$ for a GHZ state, and $$\xi _{\textrm{AME}} = 1-\frac{1}{2^N}\sum _{j=0}^N \left( {\begin{array}{c}N\\ j\end{array}}\right) \frac{1}{2^{\min (j, N-j)}}$$ for an absolutely maximally entangled (AME) state^64,65^. The top panels in Fig. 6 display the results for star, ring, and squeezing ansatz, from left to right, at $N = 4$ and (2-2) layers of each ansatz as an example. All the ansatzes examined can reach the separable and GHZ state, but hard to achieve the AME state. This observation is consistent with the CEs for conventional graph states^65^.

We next discuss the role of entanglement in achieving the ultimate SLD quantum bound. In the bottom panels of Fig. 6, we graph the corresponding CEs at the optimal CRBs shown in Fig. 3, which apparently do not require the maximum entanglement (e.g., GHZ) to achieve the ultimate SLD quantum bound. This phenomenon can be explained by the fact that maximum entanglement is not required for high-precision quantum metrology, as previously noted in Refs.^66–68^. Therefore, emphasizing the robustness of easily preparable entangled probe states and non-local POVM schemes would be advantageous for quantum metrological applications exposed to Markovian and non-Markovian noises.

### Cost functions

**Figure 7:**
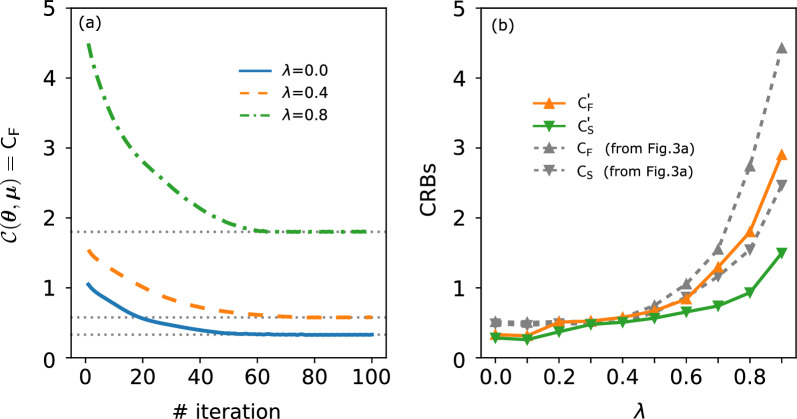
Optimizing classical bound $\textsf{C}_{\textsf{F}}$ through Variational Quantum Metrology. (**a**) Plot of the cost function $\mathcal {C} (\varvec{\theta },\varvec{\mu }) = \textsf{C}_{\textsf{F}}$ versus the number of iterations across various noise probabilities $\lambda$. (**b**) Plot of the corresponding optimal values of $\mathsf {C'}_{\textsf{F}}$ and $\mathsf {C'}_{\textsf{S}}$, and their counterparts extracted from Fig. 3a (in grayscale). These numerical results pertain to the star-graph configuration.

We address the selection of the cost function used in the variational algorithm. The preference outlined in Eq. (2) is not the sole option. An alternative approach could involve adopting the classical bound $\textsf{C}_{\textsf{F}}$ as the cost function to maximize the information extraction. However, this way does not guarantee the classical bound can reach the quantum bound, a requirement in estimation theory. In Fig. 7a, we present a plot depicting the cost function $\mathcal {C}(\varvec{\theta },\varvec{\mu })=\textsf{C}_{\textsf{F}}$ as a function of the number of iterations, considering various noise probabilities $\lambda$. It demonstrates that the cost function reaches its minimum value at a certain iteration. Correspondingly, in Fig. 7b, we provide the optimal values for both classical and quantum bounds, denoted as $\mathsf {C'}_{\textsf{F}}$ and $\mathsf {C'}_{\textsf{S}}$, alongside this optimization. It’s important to emphasize that this approach does not guarantee that $\mathsf {C'}_{\textsf{F}}$ equals $\mathsf {C'}_{\textsf{S}}$. For comparison, we include a grayscale representation of these quantities, originally presented in Fig. 3a. Here, optimizing the cost function Eq. (2) still ensures small values and convergence for both $\textsf{C}_{\textsf{F}}$ and $\textsf{C}_{\textsf{S}}$. However, $\mathsf {C'}_{\textsf{F}}$ and $\mathsf {C'}_{\textsf{S}}$ consistently remain below $\textsf{C}_{\textsf{F}}$ and $\textsf{C}_{\textsf{S}}$. This behavior occurs because the evaluation of $\textsf{C}_{\textsf{F}}$ and $\textsf{C}_{\textsf{S}}$ is based on the state that maximizes the figure of merit in Eq. (2), rather than solely minimizing $\textsf{C}_{\textsf{F}}$ or $\textsf{C}_{\textsf{S}}$.

Furthermore, alternative physical quantities, such as the tradeoff $\mathcal {T}$ and the norm-2, can also be utilized as potential cost functions. For instance, a tradeoff cost function is analogous to the one presented in Eq. (2), taking the form:

8 $$\begin{aligned} \mathcal {C}(\varvec{\theta },\varvec{\mu }) = 1 - \dfrac{1}{d} \textrm{Tr}[FQ^{-1}], \end{aligned}$$

where *d* is the number of estimated parameters. Notably, in scenarios where *Q* is a diagonal matrix and $\mathcal {C}(\varvec{\theta },\varvec{\mu }) = 0$, it results in a zero tradeoff, i.e., $\frac{F_{11}}{Q_{11}} =\cdots = \frac{F_{dd}}{Q_{dd}} = 1$. Additionally, norm-2 can also function as a viable cost function^27^.

9 $$\begin{aligned} \mathcal {C}(\varvec{\theta },\varvec{\mu }) = ||F-Q||_2, \end{aligned}$$

where $||A||_2 = \sqrt{\lambda _{\max }(A^*A)}$ represents the norm-2, with $\lambda _{\max }$ is the maximum eigenvalue. However, it is worth noting that these alternate cost functions might not be convex nor trainable^60^. As a result, the selection of the cost function in Eq. (2) is indeed appropriate.

## Methods

### Quantum circuit training

In numerical simulations, we employ the ADAM optimizer to train the VQM variables^69^, where the variables at step $k+1$ are given by

10 $$\begin{aligned} \varvec{\theta }^{k+1}=\varvec{\theta }^{k} -\alpha \frac{\hat{m}_{k}}{\sqrt{\hat{v}_{k}} + \epsilon }, \end{aligned}$$

where $m_{k}=\beta _{1} m_{k-1} +\left( 1-\beta _{1}\right) \nabla _{\varvec{\theta }}\mathcal {C}(\varvec{\theta }), v_{k}=\beta _{2} v_{k-1}+(1-\beta _{2}) \nabla _{\varvec{\theta }}^2\mathcal {C}(\varvec{\theta }), \hat{m}_{k}=m_{k} /\left( 1-\beta _{1}^{k}\right) , \hat{v}_{k}=v_{k} /\left( 1-\beta _{2}^{k}\right) ,$ with the hyper-parameters are chosen as $\alpha = 0.2, \beta _1 = 0.8, \beta _2 = 0.999$ and $\epsilon = 10^{-8}$. The gradient $\partial _{\theta _i}\mathcal {C}(\varvec{\theta })$ is given through the parameter-shift rule^70,71^. The simulations are performed in Qiskit Aer simulator^72^. The number of iterations is chosen using the EarlyStopping callback^57^.

**Figure 8:**
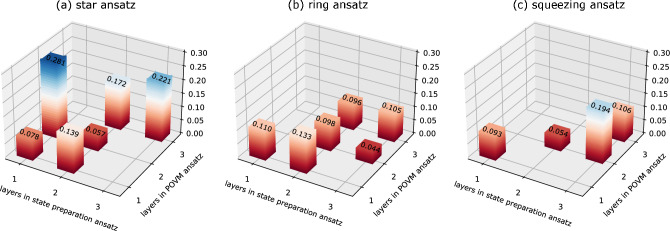
Cost function versus the optimal number of layers for different ansatzes. The plot of the cost function for (**a**) star ansatz, (**b**) ring ansatz, and (**c**) squeezing ansatz at $N = 3$ without noise. The minimum cost function vs the number of layers are (0.057, 2-2), (0.044, 3-2), and (0.054, 2-2), respectively.

To determine the appropriate number of layers for the preparation state and POVM ansatzes, we analyze the cost function (2) with different number of layers. We use $(\star ,\dagger$-$\ddagger )$ to denote the minimum cost function, the number of layers for variational state preparation, and the number of layers for variational POVM. The results are shown in Fig. 8 with $(\star ,\dagger$-$\ddagger )=$ (0.057, 2-2), (0.04, 3-2), and (0.054, 2-2) for the star, ring, and squeezing ansatz, respectively. Obviously, the metrological performances of these ansatzes demonstrate that deep ansatzes are unnecessary, as also noted in^46^ where a shallow ansatz was able to saturate quantum bound. For the numerical simulations presented in this paper, we keep the number of layers fixed at these values.

### Computing Fisher information

Classical and quantum Fisher information matrices can be computed in quantum circuits using the finite difference approximation. For the CFIM, we first derive an output probability as $\partial _{\phi _i} p = \frac{p(\phi _i + s) - p(\phi _i - s)}{2s},$ for a small shift *s*. We then compute the CFIM from $F_{ij} = \sum _m \frac{1}{p(m|\varvec{\phi })} [\partial _{\phi _i} p(m|\varvec{\phi })] [\partial _{\phi _j} p(m|\varvec{\phi })]$. For the QFIM, we explicitly derive $Q_{ij} = 2\textrm{vec} [\partial _{\phi _i}\rho (\varvec{\phi })]^\dagger \big [{\rho (\varvec{\phi })}^*\otimes \varvec{I}+\varvec{I}\otimes \rho (\varvec{\phi })\big ]^+ \textrm{vec}[\partial _{\phi _j}\rho (\varvec{\phi })],$ where $\textrm{vec}[\cdot ]$ is the vectorization of a matrix, and the superscript ‘+’ denotes the pseudo-inversion^73^. Again, we apply the finite difference to compute $\partial _{\phi _i} \rho = \frac{\rho (\phi _i + s) - \rho (\phi _i - s)}{2s},$ and substitute into the above equations to compute the QFIM.

### Supplementary Information

Supplementary Information.

## Data Availability

Data are available from the corresponding authors upon reasonable request.

## Code availability

All codes used to produce the findings of this study are incorporated into tqix^74,75^ and available at: https://github.com/echkon/tqix-developers. See also the Supplementary Material for tutorial codes.
