# Supplementary material for: Variational quantum metrology for multiparameter estimation under dephasing noise
Source: Sci Rep. 2023 Oct 18;13:17775. doi: 10.1038/s41598-023-44786-0 (PMC10584960; doi:10.1038/s41598-023-44786-0)
Supplement: Supplementary file 1 — Supplementary Information. [file 41598_2023_44786_MOESM1_ESM.pdf]

Supplementary Material: Variational quantum metrology for multiparameter estimation under dephasing noise

August 24, 2023

1 A tutorial code for running the variational quantum metrology

[1]:
```
import qiskit
import numpy as np
import tqix as tq
```

[2]:
```
# set inital circuit parameters
num_qubits = 3
num_layers = 2
t = 1.0
y = 0.1

# set initial parameters
phases = np.array([np.pi/6., np.pi/6., np.pi/6.])
params1 = np.ones(tq.create_num_params("star",num_qubits,num_layers))
params2 = np.ones(tq.create_num_params("star",num_qubits,num_layers))

# set intial circuits
qcir1 = [tq.star_ansatz,num_layers,params1]
qcir2 = [tq.u_phase,t,phases]
qcir3 = [tq.non_markovian,t,y]
qcir4 = [tq.star_ansatz_inv,num_layers,params2]

# input circuit
qcirs =[qcir1, qcir2, qcir3, qcir4]

# setup a model
qc = qiskit.QuantumCircuit(num_qubits, num_qubits)
model = tq.qc_add(qc, qcirs)
model.draw('mpl')
```

[2]:

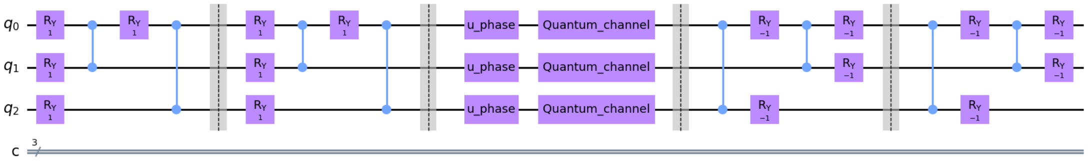

[3]:
```
# training sld bound
# allways remember reset qc

import matplotlib.pyplot as plt
num_steps = 100

qcirs, costs = tq.training(qc,
               qcirs,
               which_train = [0, 3],
               cost_func = tq.bound_sld_cls,
               grad_func = tq.grad_cost,
               opt_func = tq.adam,
               num_steps = num_steps)

# plot
iterations = list(range(1, num_steps+1))
plt.plot(iterations, np.real(costs), '-')
plt.xlim(0, num_steps)
```

```
0 0.547283631591821
1 0.5236293483540543
2 0.5028685693903935
3 0.48381577547565247
4 0.4660771875678238
5 0.44958250602492
6 0.43352177853852014
7 0.4172656619063312
8 0.4010273919478976
9 0.3852416818393688
10 0.37036761785309724
11 0.35597732195115306
12 0.3413732562539983
13 0.326522670548668
14 0.3113473639593629
15 0.29592792872375684
16 0.28044026663726496
17 0.2647956873669274
18 0.24934721580298735
19 0.2339557536061574
```

20 0.21946176145530283
21 0.2050429981336087
22 0.19141051783912844
23 0.17772095521791775
24 0.16430443595929622
25 0.15130759917743464
26 0.13871429257981638
27 0.12749409011023238
28 0.11653076284902775
29 0.10733725587101817
30 0.09861739510918988
31 0.09089210174480966
32 0.0853428497242612
33 0.07859652196602318
34 0.07344085655934085
35 0.06840468654582343
36 0.06312773837722463
37 0.06033143740958424
38 0.05629842020150633
39 0.05498758363014977
40 0.05271040435688201
41 0.05291392546978824
42 0.051108880095736664
43 0.04974539962962887
44 0.0489175040203651
45 0.048605167648577474
46 0.04680852497857291
47 0.048755906657556136
48 0.044454902611034464
49 0.04882446624964665
50 0.044311834245527204
51 0.04740456919365299
52 0.04274630875894869
53 0.04706231531553251
54 0.04265217371490859
55 0.04725219565207961
56 0.04276578660281938
57 0.04744091753757873
58 0.04287980496876198
59 0.04762851809080326
60 0.04299419191466447
61 0.047815032686848946
62 0.043108913916911296
63 0.04800049248556826
64 0.04322394023777498
65 0.04818492863487622
66 0.04333924282392221
67 0.048368367314556515

```
68 0.04345479533710961
69 0.04888992190312702
70 0.04357063675395023
71 0.04906824654256914
72 0.043686820365747336
73 0.04924554071674425
74 0.04380332080151428
75 0.04942182495620406
76 0.04392011465068846
77 0.049597122534936866
78 0.04403718009545354
79 0.049771456349528065
80 0.044154496544168254
81 0.04994484819075806
82 0.044272044811093036
83 0.05011731842120737
84 0.0443898066838011
85 0.0502888865417398
86 0.044507765319493964
87 0.05045957109144317
88 0.04462590455981186
89 0.05062938948251716
90 0.04474420919235966
91 0.05079835859704951
92 0.04486266500284364
93 0.05096649403973719
94 0.044981258281135306
95 0.05113381125230476
96 0.04509997610325711
97 0.05130032457282807
98 0.04521880648292287
99 0.051466047775567514
```

[3]: (0.0, 100.0)

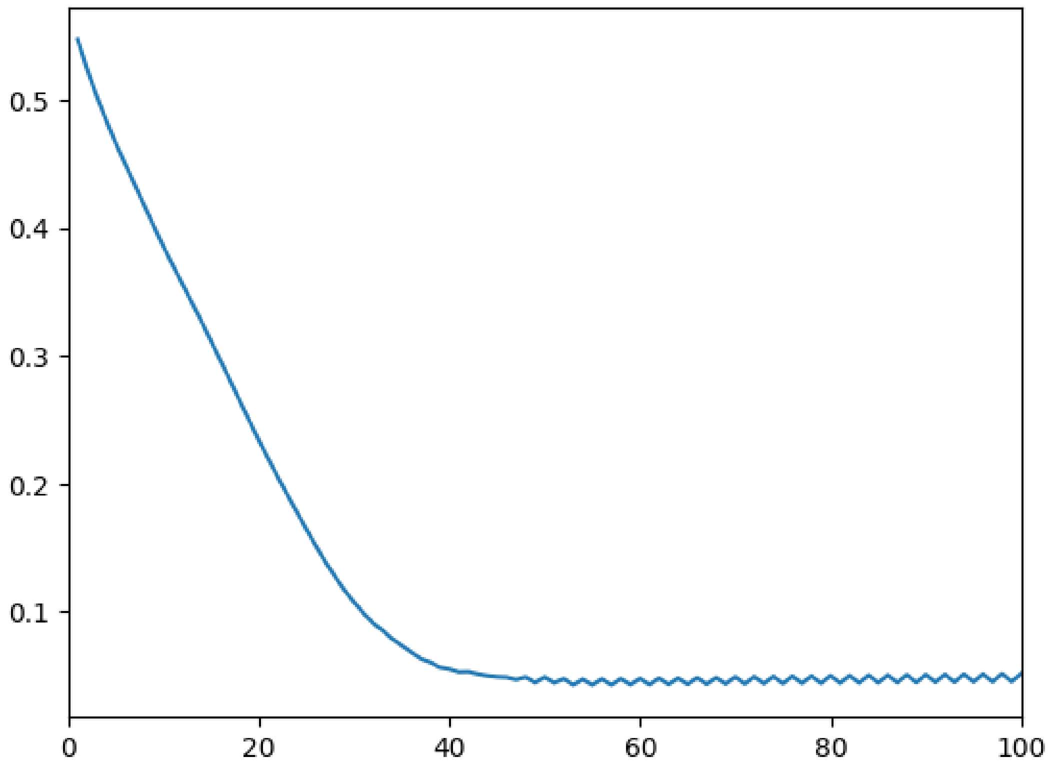


```
[4]: # quantum fisher information matrix

qc = qiskit.QuantumCircuit(num_qubits,num_qubits)
qfim = tq.sld_qfim(qc,qcirs)
cfim = tq.cfim(qc,qcirs)

# cls bound and quantum bound
cb = tq.cls_bound(qc,qcirs)
qd = tq.sld_bound(qc, qcirs)

inv_cfim = np.linalg.inv(cfim + np.eye(len(cfim)) * 10e-10)
inv_qfim = np.linalg.inv(qfim + np.eye(len(qfim)) * 10e-10)

print(cb, qd, np.trace(cfim @ inv_qfim))
```

```
0.509293974311255 0.4830826262975433 2.8325435660148934
```

2 A tutorial code for running the barren plateau

[1]:
```
import qiskit
import numpy as np
import tqix as tq
import matplotlib.pyplot as plt
```

[2]:
```
num_qubits = 3
num_layers = 2
t = 1.0
lamb = 0.1
y = tq.vqa.constants.lamb2y(t,lamb)

# set initial parameters
params1 = np.ones(tq.create_num_params("star",num_qubits,num_layers))
phases = np.array([np.pi/6., np.pi/6., np.pi/6.])
params2 = np.ones(tq.create_num_params("star",num_qubits,num_layers))

# set intial circuits
qcir1 = [tq.star_ansatz,num_layers,params1]
qcir2 = [tq.u_phase,t,phases]
qcir3 = [tq.dephasing,t,y]
qcir4 = [tq.star_ansatz_inv,num_layers,params2]

# input circuit
qcirs =[qcir1, qcir2, qcir3, qcir4]

# setup a model
qc = qiskit.QuantumCircuit(num_qubits, num_qubits)
model = tq.qc_add(qc, qcirs)
model.draw('mpl')
```

[2]:

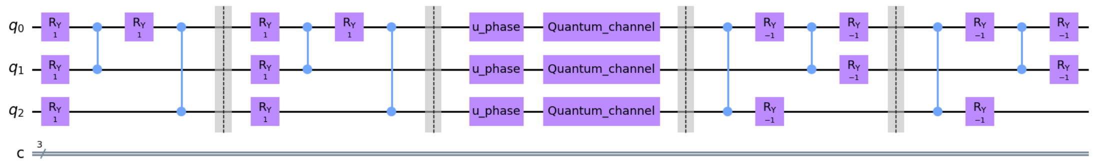

[3]:
```
#run barren plateau

nindx = np.arange(0.001, 1, 0.05).tolist()
bps = []
for i in nindx:
    y = tq.vqa.constants.lamb2y(t,i) #change lamb i
```

```
    params1 = np.random.uniform(0, 2 * np.pi, tq.
 ↪create_num_params("star",num_qubits,num_layers))
    params2 = np.random.uniform(0, 2 * np.pi, tq.
 ↪create_num_params("star",num_qubits,num_layers))

    qc = qiskit.QuantumCircuit(num_qubits, num_qubits)

    # set intial circuits
    qcir1 = [tq.star_ansatz,num_layers,params1]
    qcir2 = [tq.u_phase,t,phases]
    qcir3 = [tq.dephasing,t,y]
    qcir4 = [tq.star_ansatz_inv,num_layers,params2]

    # input circuit
    qcirs =[qcir1, qcir2, qcir3, qcir4]

    bp = tq.vqa.plateau(qc,
                        qcirs,
                        cost_func = tq.vqa.fitting.bound_sld_cls,
                        num_samples = 200)
    bps.append(np.real(bp))


# plot
plt.semilogy(nindx, bps, '.')
plt.xlim(0, 1)
```

[3]: (0.0, 1.0)

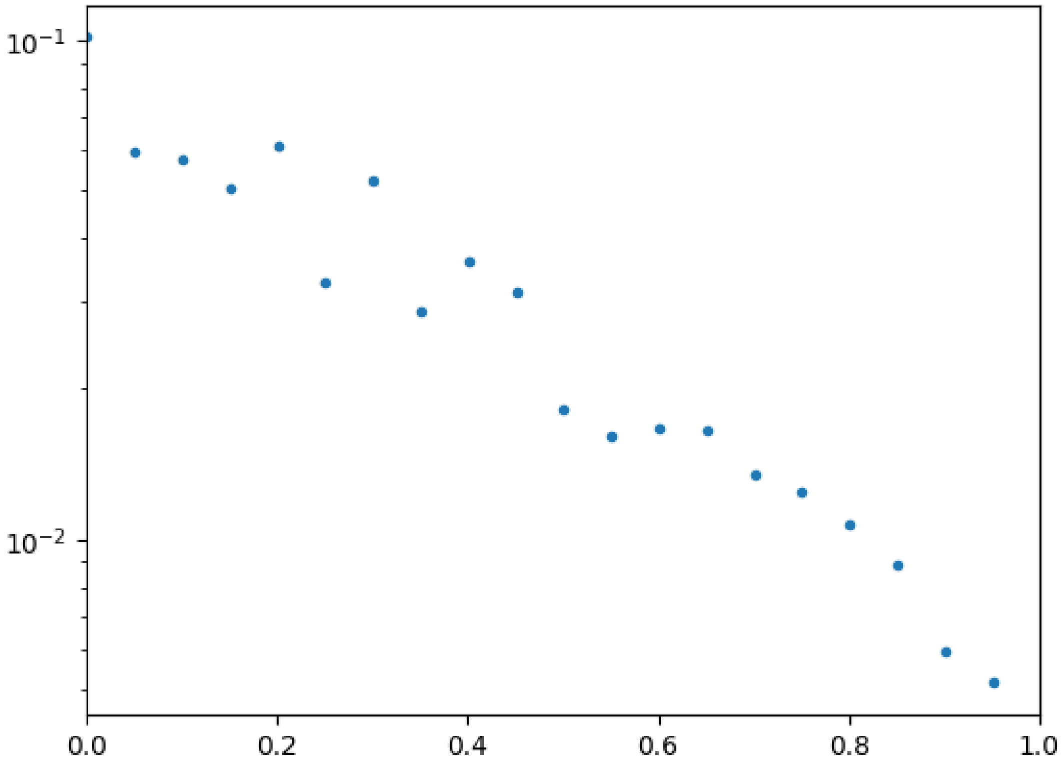

3 A tutorial code for running the concentratable entanglement

[1]:
```
import qiskit
import numpy as np
import tqix as tq
import matplotlib.pyplot as plt
```

[2]:
```
#train CE

num_qubits = 3
num_layers = 2

# set initial parameters
optimizer = tq.vqa.vqent.adam
num_steps = 100
ofset = 0.0

costs = []
ces = []

for i in range(1): #average
```

```
    params = np.random.uniform(0, 2 * np.pi, tq.
↪create_num_params("star",num_qubits,num_layers))
    qcirs = [[tq.star_ansatz,num_layers,params]]
    qc = qiskit.QuantumCircuit(num_qubits, num_qubits)

    params, cost, ce = tq.vqa.vqent.training(qc,qcirs,optimizer,num_steps,ofset)

    costs.append(cost[-1])
    ces.append(ce)

    print(i,cost[-1],ce)
```

```
0 0.006283972998339493 0.006283972998339493
```

[3]:
```
# plot
iterations = list(range(1, num_steps+1))
plt.plot(iterations, cost, '-')
plt.xlim(0, num_steps)
```

[3]: (0.0, 100.0)

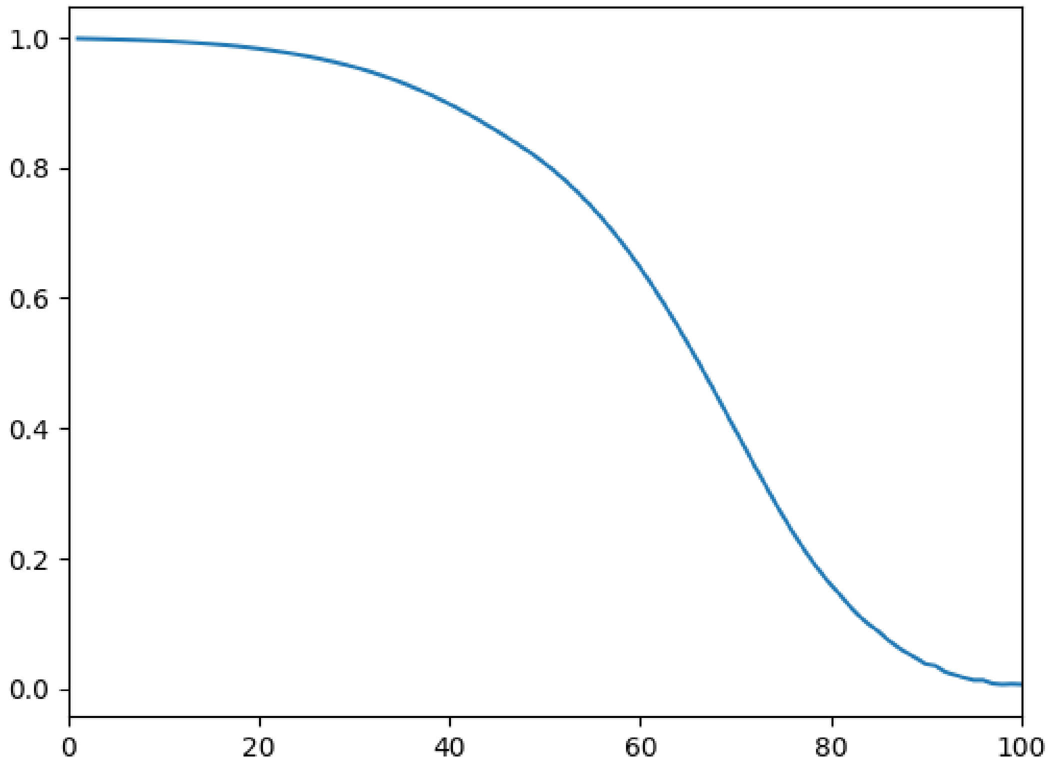

[4]:

```python
# create ghz_state
num_qubits = 3
qc = qiskit.QuantumCircuit(num_qubits, num_qubits)
qc = tq.vqa.circuits.ghz_cir(qc, None, None)
qc.draw('mpl')
```

[4]:

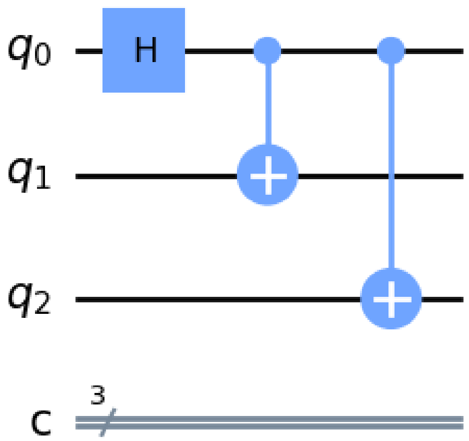

[5]:

```python
# calcualte concentratable_entanglement
ce = tq.vqa.entanglement.concentratable_entanglement(qc)
print(ce)
print("true CE = ", 0.5 - 1/(2**num_qubits))
```

```
0.36460000000000004
true CE =  0.375
```
